# Analysis of factors affecting the efficacy of contralateral seventh cervical nerve transfer for central upper limb paralysis and prediction model for efficacy evaluation

**DOI:** 10.1097/MD.0000000000046837

**Published:** 2025-12-26

**Authors:** Jiabo Xiao, Bo Zhang, Shichang Guo, Xiaohui Liu, Binhong Li, Changzheng Dong

**Affiliations:** a Department of Neurosurgery II, Hebei General Hospital, Shijiazhuang, Hebei Province, China; b Department of Postgraduate, Hebei Medical University, Shijiazhuang, Hebei Province, China; c Department of Postgraduate, Hebei North University, Zhangjiakou, Hebei Province, China.

**Keywords:** central hemiplegia, contralateral cervical nerve transposition, neural plasticity, predictive model, rehabilitation

## Abstract

This study aims to evaluate the efficacy, safety, and predictors of contralateral C7 nerve transfer (CC7) for central upper limb spastic hemiplegia and develop a preoperative prediction model. A retrospective analysis included 58 stroke patients with spastic hemiplegia undergoing CC7 surgery. Composite efficacy endpoints assessed efficacy. Univariate and multivariate logistic regression identified predictive factors. A nomogram prediction model was built and validated using receiver operating characteristic curves, calibration curves, and decision curve analysis. CC7 significantly improved affected upper limb function. The Fugl-Meyer Assessment for upper extremity (FMA-UE) score increased from 25.9 ± 3.6 preoperatively to 37.6 ± 3.4 at final follow-up (*P* < .001). Based on composite endpoints, the overall efficacy rate was 60.3% (35/58). Analysis of 15 potential factors identified 2 significant independent predictors of efficacy: younger age (odds ratio = 0.86, 95% confidence interval [CI]: 0.77–0.96, *P* = .006) and higher baseline FMA-UE score (odds ratio = 1.45, 95% CI: 1.12–1.89, *P* = .005). A nomogram incorporating these factors demonstrated strong diagnostic performance (area under the curve = 0.898, 95% CI: 0.820–0.976, *P* < .05). Bootstrap validation (area under the curve = 0.884), calibration curves, and decision curve analysis confirmed the model’s robustness and clinical utility. CC7 showed good safety; postoperative adverse reactions (transient contralateral limb numbness/pain) were mild and resolved without long-term issues. CC7 is a safe and effective treatment for central hemiplegia, significantly improving upper limb function. The developed nomogram, using age and baseline FMA-UE score, provides an accurate tool for predicting CC7 efficacy and aiding patient selection.

## 1. Introduction

Spastic hemiplegia is a common sequela following damage to the central nervous system, such as from stroke, traumatic brain injury, or autoimmune diseases.^[1]^ Globally, ~65% to 75% of stroke patients experience upper limb dysfunction, significantly impairing quality of life and work capacity.^[2,3]^ In China, over 2.5 million new stroke cases occur annually, with ~80% of survivors exhibiting varying degrees of upper limb dysfunction.^[4]^ While traditional rehabilitation therapy can partially improve motor function, its efficacy is limited for patients with severe upper limb spasticity and loss of voluntary movement.^[5–10]^ In recent years, the contralateral seventh cervical nerve transfer (CC7) procedure, based on the theory of neural plasticity, has emerged as a new treatment option for such patients.^[11–13]^ This procedure involves transferring the healthy side’s seventh cervical nerve root to the affected side, leveraging the central nervous system’s transhemispheric reorganization mechanism to reconstruct motor pathways. Clinical studies have confirmed that it can significantly improve affected limb function.^[14–16]^ However, the efficacy of CC7 varies significantly among individuals. Additionally, existing studies are largely limited to small-sample efficacy observations^[17–19]^ and have not established a systematic framework for analyzing influencing factors or predictive models.

In this study, we assessed the efficacy and safety of CC7 using the Fugl-Meyer score, Ashworth muscle tone grading, and adverse reactions. We then explored potential factors predictive of CC7 efficacy and attempted to establish a predictive model for CC7 treatment of upper limb spastic hemiplegia, aiming to provide evidence for clinical patient selection and optimization of management strategies.

## 2. Materials and methods

### 2.1. Study population and design

The study included 58 patients with spastic hemiplegia who underwent contralateral cervical seventh nerve transposition surgery (CC7) at the Department of Neurosurgery II, Hebei General Hospital, between January 2023 and March 2025. All enrolled patients had reached a plateau in their recovery, demonstrating no significant functional improvement after a minimum of 6 months of standardized conventional rehabilitation therapy prior to surgery, thereby minimizing the likelihood that observed gains were due to spontaneous recovery or standard care alone. The study integrated their baseline clinical data (demographic characteristics, stroke etiology, disease duration), preoperative functional assessments (affected upper limb Fugl-Meyer score, Ashworth muscle tone grading), cranial CT, brachial plexus MRI imaging data, intraoperative neurophysiological monitoring results, and postoperative rehabilitation logs. All patients underwent standardized preoperative assessment, CC7 surgery, and regular postoperative follow-up. Treatment efficacy was comprehensively evaluated based on dynamic assessments of motor function in the affected limb (improvement rate in Fugl-Meyer Assessment for upper extremity [FMA-UE] scores) and spasticity status (modified Ashworth scale). All study participants met the following inclusion and exclusion criteria. Inclusion criteria are as follows: clear clinical diagnosis: meeting the diagnostic criteria for spastic hemiplegia established by the World Stroke Organization^[20]^; severe unilateral upper limb dysfunction: 20 points ≤ affected upper limb Fugl-Meyer score (FMA-UE) ≤ 30 points; disease duration: onset time ranging from 6 months to 5 years, ensuring the potential for neural plasticity and the postoperative rehabilitation window period^[21]^; ineffectiveness of conservative treatment: no significant improvement in function after standardized rehabilitation therapy (≥6 months) or oral ant spasticity medication; and age and compliance: age between 18 and 75 years, conscious, and able to cooperate with postoperative follow-up. Exclusion criteria are as follows: surgical contraindications: concomitant severe cardiovascular or pulmonary disease, brachial plexus injury or peripheral neuropathy on the affected side, coagulation disorders; neurological functional limitations: severe cognitive impairment or mental illness preventing assessment and rehabilitation, concomitant other central nervous system disorders; confounding factors: undergone other nerve repair surgery within the past 6 months, pregnant or lactating women, expected survival period <1 year; and incomplete follow-up data.

**Figure 1.**
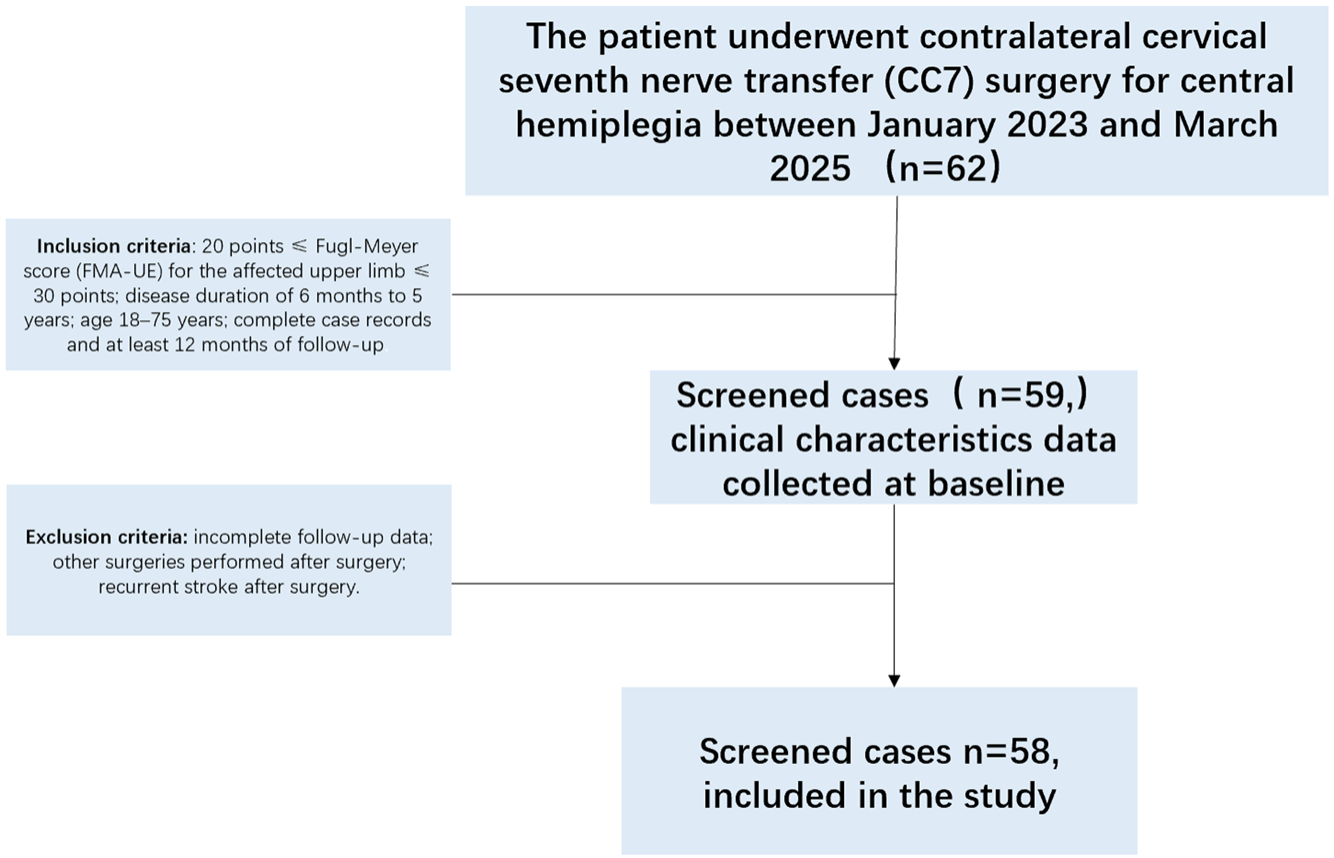
Research flowchart.

This retrospective study utilized de-identified patient data extracted from the medical records of Hebei General Hospital. The data were accessed for research purposes between January 2023 and March 2025. Authors did not have access to information that could identify individual participants during or after data collection. The requirement for written or verbal informed consent was waived by the Ethics Committee of Hebei General Hospital (Approval No. 2025-LW-0181) due to the retrospective nature of this study. All patient data were anonymized and de-identified prior to analysis. Figure 1 illustrates the patient selection flowchart.

All patients underwent a comprehensive preoperative assessment, including comprehensive blood tests (liver and kidney function assessment, coagulation function testing, biochemical indicator analysis, etc) to determine the patient’s tolerance for surgery. Cranial MRI/CT: to identify the location (e.g., basal ganglia, corticospinal tract) and extent of brain injury and rule out new lesions. Brachial plexus MRI: observe the course, diameter, and structural abnormalities of the contralateral C7 nerve to guide intraoperative localization.

To accurately quantify the severity of central hemiplegia in patients, all patients completed the FMA-UE and the Modified Ashworth Scale (MAS) within 1 week prior to surgery. The FMA-UE is the upper limb-specific component of the FMA, comprising 33 items with a maximum score of 66 points, using a 3-point scoring system (Table 1). The MAS assesses spasticity (muscle tone) using a 0 to 5 point scale, with higher scores indicating more severe spasticity.

**Table 1:** Baseline data for 58 patients with central hemiplegia.

Variable	Overall (n = 58)	Effective group (n = 35)	Ineffective group (n = 23)
Sex (no., %)			
Male	36 (62.1)	23 (65.7)	13 (56.5)
Female	22 (37.9)	12 (34.3)	10 (43.5)
age (yr)	52.2 ± 11.0	47.1 ± 10.1	59.6 ± 7.0
BMI (kg/m^2^)	26.2 ± 3.3	26.5 ± 3.4	25.7 ± 3.1
During (yr)	2.7 ± 2.3	2.1 ± 1.9	3.5 ± 2.5
Rehabilitation (no., %)			
Yes	25 (43.1)	24 (68.6)	1 (4.3)
No	33 (56.9)	11 (31.4)	22 (95.7)
Surgery duration (h)	6.6 ± 1.9	6.9 ± 2.1	6.2 ± 1.5
Affected side (no., %)			
Left	34 (58.6)	22 (62.9)	12 (52.2)
Right	24 (41.4)	13 (37.1)	11 (47.8)
Causes (no., %)			
Hemorrhagic stroke	31 (53.4)	21 (60.0)	10 (43.5)
Ischemic stroke	27 (46.6)	14 (40.0)	13 (56.5)
Combined (no., %)			
Diabetes	19 (32.8)	13 (37.1)	6 (26.1)
Hypertension	41 (70.7)	26 (74.2)	15 (65.2)
Coronary heart disease	5 (8.6)	1 (2.9)	4 (17.4)
Epilepsy	4 (6.9)	3 (8.6)	1 (4.3)
*DA nerves (no., %)			
Yes	49 (84.5)	34 (97.1)	15 (65.2)
No	9 (15.5)	1 (2.9)	8 (34.8)
Previous surgery (no., %)			
Yes	21 (36.2)	15 (42.9)	6 (26.1)
No	37 (63.8)	20 (57.1)	17 (73.9)
Fugl-Meyer score	25.9 ± 3.6	27.4 ± 3.0	25.6 ± 2.9
Modified Ashworth Scale			
Forearm	0 (1), 1 (26), 2 (28), 3 (3)	0 (0), 1 (12), 2 (20), 3 (3)	0 (1), 1 (14), 2 (8)
Elbow	0 (0), 1 (18), 2 (24), 3 (16)	0 (0), 1 (12), 2 (16), 3 (7)	0 (0), 1 (6), 2 (8), 3 (9)
Wrist	0 (0), 1 (3),2 (17), 3 (28)	0 (0), 1 (2),2 (13), 3 (20)	0 (0), 4 (1),2 (4), 3 (8)
Thumb	0 (0), 1 (22), 2 (28), 3 (8)	0 (0), 1 (14), 2 (18), 3 (3)	0 (0), 1 (8), 2 (10), 3 (5)
Fingers 2–5	0 (0), 1 (28), 2 (26), 3 (4)	0 (0), 1 (16), 2 (16), 3 (3)	0 (0), 1 (12), 2 (10), 3 (1)

Data are presented as mean ± SD unless otherwise specified. The Fugl-Meyer Upper Limb Scale is used to assess motor dysfunction, with a scoring range of 0 to 66 (higher scores indicate better function). The Modified Ashworth Scale is used to measure spasticity (muscle tone) in paralyzed arms, with a scoring range of 0 to 5 for each joint (higher scores indicate more severe spasticity).

* DA nerves, decellularized allogeneic nerves.

Based on literature evidence,^[17,22]^ the following prognostic factors were selected as potential clinical indicators for the efficacy of CC7 treatment in upper limb spastic hemiplegia: gender, age, height, weight (body mass index [BMI] calculation), side of hemiplegia (left/right), stroke type (ischemic/hemorrhagic), duration of hemiplegia (years), comorbidities affecting subsequent rehabilitation (hypertension, diabetes, coronary heart disease, epilepsy, etc), prior surgical history due to underlying conditions, Fugl-Meyer Upper Extremity Score (FMA-UE, 0–66 points), intraoperative nerve anastomosis status (tension-free anastomosis/artificial nerve anastomosis), systematic rehabilitation therapy, and surgical duration.

### 2.2. CC7 surgical method

This study utilized the Huashan pre-spinal route.^[13]^ Following successful general anesthesia, the patient was positioned in the supine position. Two transverse incisions, each ~5 centimeters in length, were made on either side of the neck, along the lateral border of the sternocleidomastoid muscle and above the clavicle. After incising the skin and subcutaneous tissue, the sternocleidomastoid muscle on the healthy side was bluntly dissected. Concurrently, the external jugular vein on the healthy side was dissected and preserved. Access to the anatomical space was gained through the lateral and inferior borders of the sternocleidomastoid muscle. Soft tissues were bluntly dissected to expose the phrenic nerve and anterior scalene muscle. The C5, C6, and C7 nerve roots were carefully dissected and anatomically localized. A nerve stimulator was used during the procedure to confirm the position of the nerve roots. The C7 nerve innervates the triceps brachii, extensor carpi radialis, and extensor digitorum muscles, primarily responsible for elbow and wrist extension. When the nerve probe is placed on C7 and a 1-mA current is applied, extension movements occur in the arm, elbow, and wrist; when the nerve probe is placed on C5 and C6 and a 1-mA current is applied, flexion movements occur in the arm, elbow, and wrist. After confirming C7, dissect its distal end and transect it at the junction where its anterior and posterior branches meet other brachial plexus branches to ensure the maximum length of the contralateral C7 nerve. Transect the scalene muscle along the C7 nerve root, dissect the proximal part of the C7 nerve root to the intervertebral foramen, and take care to protect the phrenic nerve, vertebral vein, and artery. Expose the contralateral vertebral body anterior mediastinum and esophagus, and transect the sternocleidomastoid tendon. Identify and dissect the proximal portion of the affected C7 nerve root in the same manner, and transect the C7 nerve root at the intervertebral foramen. Expose the anterior esophageal cavity of the affected vertebral body and the ipsilateral longus colli muscle. Through the posterior intervertebral foramen space, transpose the contralateral C7 nerve deep to the sternocleidomastoid muscle to the affected side and anastomose it with the affected C7 nerve. For cases with excessively long anastomosis distances, decellularized allogeneic nerves (model: ZDMED-5060) can be used, followed by tension-free suturing. Postoperatively, place drainage tubes on both sides of the neck incision, apply a cervical collar for fixation, and secure the affected upper limb with an arm sling.

### 2.3. Postoperative rehabilitation

The rehabilitation program in this study clearly recommends that patients adhere to systematic, standardized rehabilitation training for at least 12 months or longer after surgery.^[23]^ However, during the study, it was observed that most patients faced significant financial pressure and were unable to afford the costs of long-term, high-frequency professional rehabilitation treatment (including treatment fees, transportation costs, lost wages, etc). Therefore, receipt of systematic rehabilitation training was recorded as a candidate predictor variable (independent variable). We acknowledge that this binary classification (yes/no) is a significant simplification that fails to capture the intensity, frequency, duration, and content of rehabilitation, which likely obscures the true dose–response relationship between rehabilitation and outcome.

### 2.4. Postoperative follow-up

All patients underwent regular postoperative follow-up and assessment at 1, 3, 6, and 12 months postoperatively, using various methods such as outpatient visits, telephone contact, WeChat, and questionnaire surveys, respectively. Follow-up assessments included the Fugl-Meyer Upper Extremity Function Score (FMA-UE, 0–66 points), the MAS (grading of shoulder, elbow, and wrist joints), and the occurrence of adverse reactions.

This study adopted a composite efficacy endpoint: the Fugl-Meyer Upper Extremity Score (FMA-UE) at 1 month preoperatively was used as the baseline score. Primary criterion: Improvement in the Fugl-Meyer Upper Extremity Score (FMA-UE) postoperatively compared with baseline ≥ 10 points. Secondary criteria: A reduction of ≥ 1 grade in the MAS score for at least 2 joints; ability to perform ≥ 3 functional tasks (dressing, tying shoelaces, wringing out a towel, using a mobile phone). Meeting both the primary criterion and at least 1 secondary criterion is considered “effective,” otherwise, it is considered “ineffective.”

### 2.5. Statistical analysis

All analysis was performed using SPSS software (version 27.0; IBM Corporation, Armonk) and R software (version 4.5.1; Statistical Computing R Foundation, Vienna, Austria). Continuous variables are expressed as mean ± standard deviation or median (interquartile range), while categorical data are presented as counts and percentages. Statistical analyses were performed using 2-tailed tests with 95% confidence intervals (CI). *P* < .05 was considered statistically significant. Intragroup comparisons were performed using paired *t* tests and paired Wilcoxon signed-rank tests. Univariate logistic regression analysis was conducted with composite efficacy endpoints as the dependent variable to identify factors potentially influencing efficacy. Based on the results of the multivariate logistic regression analysis, factors with *P* < .05 were selected to construct a nomogram predicting the efficacy of CC7 in patients with central hemiplegia. The model’s validity was evaluated using receiver operating characteristic (ROC) curves and Bootstrap models. The calibration curve was used to assess the concordance between the model’s probability curve and the ideal curve. The clinical decision curve analysis was used to evaluate the clinical benefit of our model.

## 3. Results

### 3.1. Baseline data and clinical characteristics

This study included 58 patients with central hemiplegia who underwent contralateral cervical seventh nerve transposition surgery, including 36 males (62.1%) and 22 females (37.9%). The median age of the patients was 52.2 ± 11.0 years, and the median BMI was 26.2 ± 3.3 kg/m². The distribution of affected sides was 34 cases (58.6%) on the left side and 24 cases (41.4%) on the right side. The median duration of illness was 2.7 ± 2.3 years. In terms of comorbidities, 41 patients (70.7%) had hypertension, 19 patients (32.8%) had diabetes, 5 patients (8.6%) had coronary heart disease, and 4 patients (6.9%) had epilepsy. The primary causes included hemorrhagic stroke (31 cases, 53.4%) and ischemic stroke (27 cases, 46.6%), with 21 cases (36.2%) having undergone surgical intervention for the primary disease. Preoperative functional assessment showed that upper limb muscle tone was mainly grade 2 on the Ashworth scale. The median Fugl-Meyer upper limb score was 25.9 ± 3.6 points. The median surgical duration was 6.6 ± 1.9 hours. All nerve anastomoses were end-to-end anastomoses (58 cases, 100.0%), and all tract construction utilized the prevertebral approach (58 cases, 100.0%). The coverage rate of postoperative rehabilitation interventions was 43.1% (20 cases). Baseline characteristics of the patients are detailed in Table 1.

### 3.2. Efficacy of contralateral C7 nerve transposition

#### 3.2.1. Improvement of motor function in patients with central upper limb paralysis

**Figure 2.**
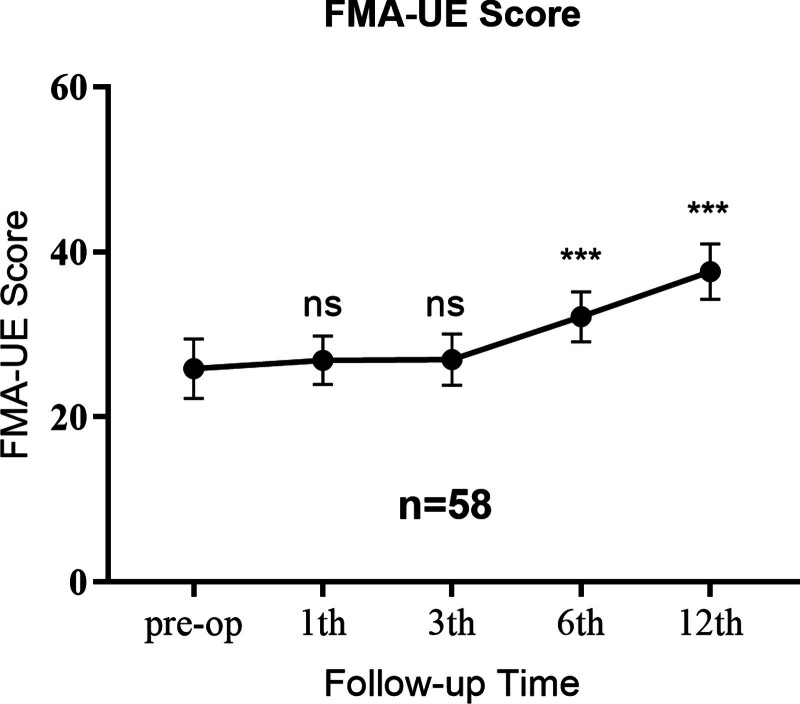
Line graph of mean ± SD Fugl-Meyer Assessment (FMA) scores before and after CC7. FMA-UE refers to upper extremity-specific part of the FMA, which consists of 33 items with a maximum score of 66, using a 3-level scoring system. Ns = not significant, SD = standard deviation. *P < .05, **P < .01, ***P < .001.

The Fugl-Meyer Upper Limb Score was used, which was completed by 2 neurosurgeons and assessed patients’ postoperative upper limb, wrist, hand, coordination/speed, and lower limb and coordination/speed from 5 dimensions. The number of patients at 1, 3, 6, and 12 months postoperatively was 58, 58, 58, and 18, respectively. The decrease in patient numbers at 12 months was solely due to the predefined data collection cutoff point for this analysis, not due to patient dropout or loss to follow-up. During the follow-up period, patients’ Fugl-Meyer upper limb scores gradually improved. We selected assessment data from 58 patients who had been followed up for more than 6 months for analysis. Paired-sample *t* tests were used to analyze the statistical differences between scores at each follow-up time point and baseline data. See Figure 2. Statistical analysis showed that there were statistically significant differences in patients’ Fugl-Meyer upper limb scores starting from 6 months postoperatively (*P* < .05).

Treatment efficacy was assessed using composite efficacy endpoints. Although 40 patients (69.0%) met the primary FMA-UE criteria, only 35 patients (60.3%) achieved the full composite endpoint. The 5 patients who did not achieve the composite endpoint showed improvement in FMA-UE scores, but the reduction in spasticity or functional improvement was insufficient.

The preoperative Fugl-Meyer total score was 25.9 ± 3.6. The Fugl-Meyer total scores at 1, 3, 6, and 12 months postoperatively were 26.9 ± 3.0, 27.0 ± 3.1, 32.1 ± 3.0, and 37.6 ± 3.4, respectively. Statistically significant differences in the Fugl-Meyer total score began to emerge at 6 months postoperatively. Nonparametric tests were used to compare baseline scores with scores at each follow-up time point. At 1 month postoperatively: the score was 26.9 ± 3.0, with no significant difference from baseline (*P* = .67); At 3 months postoperatively: the score was 27.0 ± 3.1, with no significant difference from baseline (*P* = .209); 6 months postoperatively: score 32.1 ± 3.0, an increase of 23.9% from baseline (*P* < .001); and 12 months postoperatively: score 37.6 ± 3.4, an increase of 45.2% from baseline (*P* < .001). Detailed scores are shown in Table 2. It is worth noting that 3 patients experienced a rebound at 12 months postoperatively, with Fugl-Meyer scores at 12 months postoperatively lower than those at 6 months postoperatively. The long-term outcomes based on the subset of 18 patients with 12-month data should be interpreted with caution.

**Table 2:** Fugl-Meyer scores before and after surgery.

Indicator	Baseline	1 mo (n = 58)	*P*	3 mo (n = 58)	*P*	6 mo (n = 58)	*P*	12 mo (n = 18)	*P*
Upper limb	14.5 ± 1.6	14.6 ± 1.3	.901	14.7 ± 1.4	.149	18.6 ± 1.2	<.001	20.8 ± 2.6	<.001
Wrist	4.0 ± 0.4	4.0 ± 0.3	.850	4.1 ± 0.4	.148	5.3 ± 0.3	<.001	6.2 ± 0.7	<.001
Hand	5.6 ± 0.6	5.7 ± 0.5	.880	5.7 ± 0.6	.152	7.4 ± 0.4	<.001	8.5 ± 1.0	<.001
Coordination/Speed	2.4 ± 0.3	2.4 ± 0.2	.920	2.5 ± 0.3	.145	3.1 ± 0.2	<.001	3.7 ± 0.4	<.001
Total	25.9 ± 3.6	25.9 ± 3.2	.670	26.3 ± 3.1	.209	32.1 ± 3.0	<.001	37.6 ± 3.4	<.001

All data are presented as mean ± standard deviation; the *P*-value here represents the significance of the paired-sample *t*-test comparing the scale scores at each follow-up time point with their corresponding baseline scores; the maximum scores for the upper limb, wrist, hand, and coordination/speed are 36, 10, 14, and 6 points, respectively, with a total maximum score of 66 points.

#### 3.2.2. Changes in MAS after CC7

According to MAS measurements, significant spasticity reduction was observed across all joints from baseline to 6 months postoperatively (elbow: *P* < .001; forearm: *P* < .001; wrist: *P* < .001; thumb: *P* < .001; fingers 2–5: *P* < .001). These improvements are detailed in Table 3.

**Table 3:** Modified Ashworth Scale changes from baseline to 6 months postoperatively (n = 58).

MAS	Time point	MAS grade distribution (0–4)	Change in MAS score (median, min-max)	*P*-value*
Forearm	Baseline	0 (1, 1.7%), 1 (26, 44.8%), 2 (28, 48.3%), 3 (3, 5.2%)	−1 (−2 to 0)	<.001
	6 mo	0 (8, 13.8%), 1 (32, 55.2%), 2 (18, 31.0%), 3 (0, 0%)		
Elbow	Baseline	0 (0, 0%), 1 (18, 31.0%), 2 (24, 41.4%), 3 (16, 27.6%)	−1 (−2 to 0)	<.001
	6 mo	0 (6, 10.3%), 1 (30, 51.7%), 2 (20, 34.5%), 3 (2, 3.4%)		
Wrist	Baseline	0 (0, 0%), 1 (3, 5.2%), 2 (17, 29.3%), 3 (38, 65.5%)	−1 (−3 to 0)	<.001
	6 mo	0 (10, 17.2%), 1 (20, 34.5%), 2 (25, 43.1%), 3 (3, 5.2%)		
Thumb	Baseline	0 (0, 0%), 1 (22, 37.9%), 2 (28, 48.3%), 3 (8, 13.8%)	−1 (−2 to 0)	<.001
	6 mo	0 (8, 13.8%), 1 (37, 63.8%), 2 (13, 22.4%), 3 (0, 0%)		
Fingers 2–5	Baseline	0 (0, 0%), 1 (28, 48.3%), 2 (26, 44.8%), 3 (4, 6.9%)	−1 (−2 to 0)	<.001
	6 mo	0 (15, 25.9%), 1 (35, 60.3%), 2 (8, 13.8%), 3 (0, 0%)		

MAS = Modified Ashworth Scale, max = maximum, min = minimum.

* *P*-value: baseline versus 6 months paired Wilcoxon test. Percentage calculation: Number of people in each grade/total number of people (58) × 100%. Change value calculation: 6-month score − baseline score (negative values indicate reduced spasticity).

### 3.3. Predictors and predictive models for CC7 efficacy

#### 3.3.1. Factors influencing CC7 efficacy

Based on preoperative assessments, baseline data, literature reviews, and other materials, 15 factors that may influence the efficacy of CC7 were identified, including gender (male/female), age (years), BMI, affected limb (left/right), history of diabetes, history of coronary heart disease, history of hypertension, history of epilepsy, type of underlying condition causing hemiplegia (hemorrhagic/ischemic), use of artificial nerves during surgery (yes/no), duration of hemiplegia (years), history of surgery for the underlying disease, baseline Fugl-Meyer upper limb score, duration of surgery (hours), and postoperative systematic rehabilitation.

Table 4 presents the results of univariate and multivariate logistic regression analyses of factors associated with the efficacy of CC7. Univariate analysis revealed that younger age (odds ratio [OR] = 0.84, 95% CI: 0.77–0.93, *P* < .001), shorter disease duration (OR = 0.75, 95% CI: 0.57–0.99, *P* = .041), The use of decellularized allogeneic nerves (OR = 0.07, 95% CI: 0.01–0.59, *P* = .015) and higher baseline Fugl-Meyer scores (OR = 1.45, 95% CI: 1.19–1.78, *P* < .001) were significantly associated with treatment outcomes. After including variables with *P* < .05 in the univariate analysis in the multivariate analysis, only age (OR = 0.86, 95% CI: 0.77–0.96, *P* = .006) and Fugl-Meyer score (OR = 1.45, 95% CI: 1.12–1.89, *P* = .005) remained independent prognostic factors.

**Table 4:** Results of univariate and multivariate logistic regression analysis showing prognostic factors of CC7.

Variable	Univariate analysis OR (95% CI)	*P*-value	Multivariate analysis OR (95% CI)	*P*-value
Sex (male vs female)	1.47 (0.50–4.34)	.481	-	-
Age (yr)	0.84 (0.77–0.93)	<.001	0.86 (0.77–0.96)	.006
BMI (kg/m^2^)	1.09 (0.92–1.29)	.333	-	-
Affected side (left vs right)	1.55 (0.53–4.51)	.420	-	-
Diabetes (yes vs no)	1.67 (0.53–5.32)	.382	-	-
Hypertension (yes vs no)	1.54 (0.49–4.84)	.459	-	-
Coronary heart disease (yes vs no)	0.14 (0.01–1.34)	.088	-	-
Epilepsy (yes vs no)	2.06 (0.20–21.14)	.542	-	-
Type of stroke (hemorrhagic/ischemic)	1.95 (0.67–5.66)	.220	-	-
Course of disease (yr)	0.75 (0.57–0.99)	.041	0.76 (0.50–1.14)	.187
DA nerves (yes vs no)	0.07 (0.01–0.59)	.015	0.06 (0.00–2.12)	.122
Primary disease requires surgery (yes vs no)	2.12 (0.68–6.69)	.198	-	-
Operative time (h)	1.27 (0.91–1.77)	.162	-	-
Fugl-Meyer score	1.45 (1.19–1.78)	<.001	1.45 (1.12–1.89)	.005
Systemic rehabilitation (yes vs no)	2.70 (0.82–8.93)	.104	-	-

BMI = body mass index, CC7 = contralateral cervical seventh nerve transfer, DA nerves = decellularized allogeneic nerves, OR = odd ratio.

#### 3.3.2. Nomogram for CC7 validity

**Figure 3.**
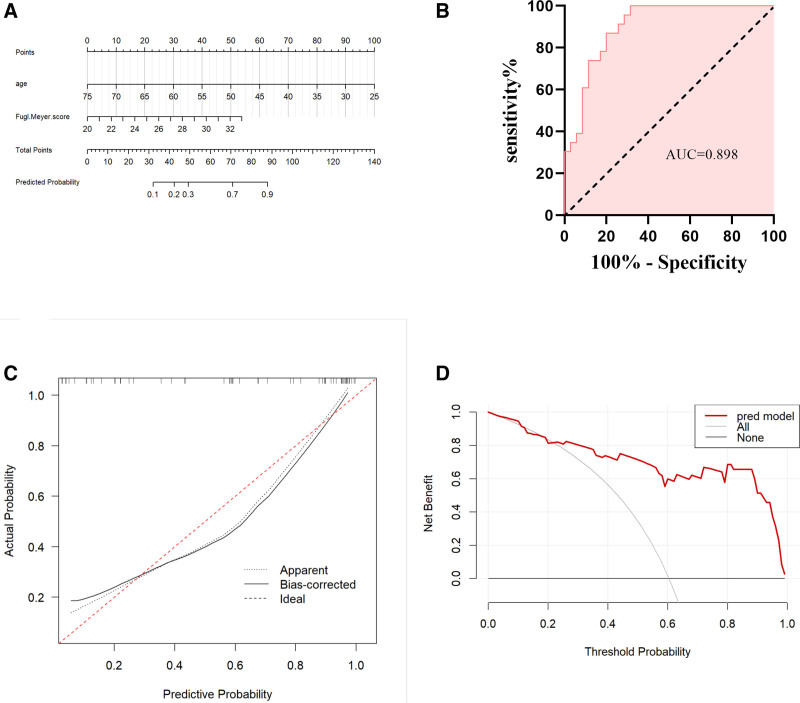
The nomogram model predicts the probability of CC7 being effective in patients with hemiplegia after stroke. (A) Validation of the predictive nomogram model. Receiver operating characteristic curve. (B) Calibration curve for the predicted probability of nomogram, with the *x*-axis representing the predicted probability and the *y*-axis the actual observed probability. (C) Clinical decision curve for the predictive model. (D) AUC = area under the receiver operating characteristic curve.

This study included 15 clinical factors that may influence the efficacy of CC7 therapy, ultimately identifying 2 variables: age and baseline Fugl-Meyer score. Based on the aforementioned logistic regression analysis, a predictive model for the efficacy of CC7 treatment in patients with central hemiplegia was constructed: Logit(P) = 0.272 + 0.375 × [Fugl-Meyer score] − 0.154 × [age (years)]. The model fit was good as indicated by the Hosmer–Lemeshow test (χ² = 5.9715, *P* = .6504). The probability of effective treatment in patients with spastic hemiplegia of the upper limb after CC7 can be estimated using a nomogram, as shown in Figure 3A.

#### 3.3.3. Model validation

The performance of the column chart was validated using ROC curve analysis, which yielded an area under the ROC curve (AUC) of 0.898 and a 95% CI of 0.820 to 0.976, indicating good diagnostic performance (Fig. 3B). Bootstrap analysis was performed with 1000 repetitions, and the C-index of the Bootstrap stepwise model was 0.884, similar to the performance of the initial predictive model. The calibration curve is shown in Figure 3C, indicating that the nomogram may underestimate the probability of a CC7 response for probabilities ranging from 0.3 to 0.9. Overall, the model demonstrated good fit and calibration with the ideal curve. Additionally, decision curve analysis indicated that the predictive model had a favorable net benefit when the threshold probability for CC7 efficacy was 0.3 to 0.9, suggesting that the model has potentially beneficial clinical implications (Fig. 3D).

### 3.4. Adverse reactions of CC7

The postoperative complications of CC7 are mainly transient sensory-motor disorders, and the overall safety is good. In this study of 58 patients, 55 (94.8%) experienced perioperative adverse reactions, all of which were reversible, with no cases of nerve graft rejection or permanent functional impairment. The main complications manifested as transient functional disorders of the contralateral upper limb: Sensory abnormalities: 94.8% (55/58) of patients experienced numbness in the unaffected limb 1 month postoperatively, with 10.3% (6/58) persisting until 3 months postoperatively, and all symptoms resolved completely by 6 months; Motor dysfunction: 31.0% (18/58) of patients had decreased muscle strength (unaffected side) 1 month postoperatively, with 3.4% (2/58) persisting until 3 months postoperatively; and Reduced sensory sensitivity: 37.9% (22/58) had increased tactile thresholds, and 34.5% (20/58) had impaired 2-point discrimination, both of which resolved within 6 months postoperatively. The incidence of surgery-related complications was low and self-limiting: 3.4% (2/58) developed subcutaneous hematomas, which resolved spontaneously without intervention; 1.7% (1/58) developed Grade A chyle leakage (mild lymphatic fluid leakage), which resolved with conservative treatment; and secondary symptoms included postoperative pain (25.9%, 15/58), fatigue (20.7%, 12/58), and a sensation of foreign body in the throat (8.6%, 5/58), all of which resolved within 3 months postoperatively. Time distribution characteristics showed: the first month postoperatively was the peak period for complications (100% of patients experienced ≥ 1 symptom); the overall incidence rate decreased to 13.8% (8/58) by 3 months postoperatively; and all complications had completely resolved by 6 months postoperatively. Thus, complications associated with the CC7 procedure primarily involve transient neurological dysfunction on the unaffected side (94.8%), which is a physiological response expected from the surgery. Severe surgery-related complications are rare (≤3.4%) and self-resolving, confirming the clinical safety of the procedure. The detailed distribution of adverse events over time is summarized in Table 5.

**Table 5:** Adverse reactions following CC7.

Event type	1 Month (n = 58)	3 Months (n = 58)	6 Months (n = 58)	12 Months (n = 18)
Any	58 (100%)	8 (13.8%)	0	0
Bleeding	0	0	0	0
Infection	0	0	0	0
Pain	15 (25.9%)	2 (3.4%)	0	0
Foreign body sensation in swallowing	5 (8.6%)	0	0	0
Fatigue	12 (20.7%)	1 (1.7%)	0	0
Numbness (contralateral side)	55 (94.8%)	6 (10.3%)	0	0
Decreased muscle strength (contralateral side)	18 (31.0%)	2 (3.4%)	0	0
Increased tactile threshold (contralateral side)	22 (37.9%)	3 (5.2%)	0	0
Subcutaneous hematoma	2 (3.4%)	0	0	0
Grade A chylous leakage	1 (1.7%)	0	0	0

Data are presented as number (percentage).

## 4. Discussion

This study is the first to establish a nomogram model for predicting the efficacy of contralateral cervical seventh nerve transposition surgery (CC7) in treating spastic hemiplegia of the upper limb following stroke. Central to our findings is the 60.3% efficacy rate based on a rigorous composite endpoint, surpassing conventional single, metric assessments by requiring concurrent improvement in motor function (≥10-point FMA-UE gain), spasticity reduction (≥1 MAS grade in ≥ 2 joints), AND/OR functional recovery (≥3 tasks). This multidimensional criterion explains the observed discrepancy between the 69.0% FMA-UE response rate and final 60.3% efficacy rate, highlighting that isolated motor improvement may not translate to functional utility. Fugl-Meyer Upper Extremity Assessment Scale (FMA-UE) score improved significantly from 25.9 ± 3.6 preoperatively to 37.6 ± 3.4 postoperatively, *P* < .001. All surgery-related complications were transient and resolved completely within 6 months postoperatively, further confirming the safety of this surgical procedure. Importantly, through multivariate regression analysis, we established age (OR = 0.86, 95% CI: 0.77–0.96, *P* = .006) and baseline FMA-UE score (OR = 1.45, 95% CI: 1.12–1.89, *P* = .005) as independent predictors of efficacy. The predictive model established based on these findings demonstrated excellent discriminative ability (AUC = 0.898) and good calibration. These findings provide strong evidence-based support for clinicians to screen suitable patients preoperatively and optimize perioperative decision-making.

The surgical outcomes following CC7 transfer are primarily categorized into short- and long-term results.^[24,25]^ In this study, the significant improvement in FMA-UE scores (an increase of 23.9% from baseline) began at 6 months post-surgery, which aligns with the previously reported time window for neural regeneration and functional reorganization in prior studies.^[26,27]^ The MAS score showed a downward trend in the early postoperative period, suggesting that the relief of spasticity may precede the significant recovery of active motor function. This is consistent with previous studies.^[11,17,19]^ However, 3 patients exhibited a rebound in FMA-UE scores at the 12-month postoperative follow-up, which may be related to interrupted rehabilitation therapy or the entry into a plateau phase of neural functional compensation. Further observation is required in long-term follow-up. In terms of safety, this study observed transient sensory disturbances in the contralateral (donor limb) in 94.8% of patients postoperatively, but all symptoms resolved completely within 6 months, with no vascular injury or permanent nerve damage occurring. This result is consistent with multiple studies,^[28–30]^ fully confirming that CC7 surgery has controllable risks and a favorable risk-benefit ratio.

In this study, age was confirmed to be a significant negative predictor of treatment efficacy. As age increases, the decline in neuroplasticity and reduced axonal regeneration capacity are well-established biological phenomena.^[31]^ In this study, we found that efficacy was significantly reduced in patients over 60 years of age, suggesting that there may be an age-related threshold for brain functional remodeling. This finding differs from the report by Luo W et al., who stated that “patients over 45 years of age did not benefit differently from those under 45 years of age.”^[32]^ This discrepancy may be attributed to the inclusion of a higher average age (52.2 ± 11.0 years) in the study.

In this study, baseline FMA-UE scores had positive predictive value. Higher baseline scores reflected relatively intact residual corticospinal tract fibers,^[33,34]^ which provided the necessary anatomical and physiological basis for functional reorganization of neural pathways. This pattern is consistent with the widely observed finding in the field of neurological rehabilitation that baseline functional status is a key factor influencing treatment response.^[35]^

Rehabilitation demonstrated a strong influence on treatment efficacy in this study. Although it failed to emerge as an independent predictor in the multivariable logistic regression analysis (OR = 2.70, *P* = .104), the response rate in the rehabilitation group (68.6%) was significantly higher than that in the non-rehabilitation group (4.3%, *P* < .001). This seemingly contradictory phenomenon primarily stems from the limitations of the study methodology: constrained by patients’ economic burdens (56.9% were unable to sustain professional rehabilitation), rehabilitation adherence was simplified into a binary variable (yes/no), failing to precisely quantify training intensity, frequency, duration, and content.

Univariate logistic regression analysis showed that the use of decellularized allogeneic nerves during surgery due to excessive anastomosis length (9 patients, 8 of whom had poor outcomes) was associated with treatment efficacy. The study found that axonal regeneration occurs at a rate of only 1 to 2 mm/day, and regeneration efficiency drops sharply when the defect exceeds 30 mm.^[36–38]^ The use of decellularized allogeneic nerves may result in reduced nerve regeneration efficiency or delayed effects, which may be one of the reasons for its poor efficacy. Previous studies have shown that patients requiring nerve transplantation have poorer outcomes compared with those with bilateral C7 nerve direct anastomosis,^[39]^ indicating that its potential negative effects should not be overlooked. Therefore, efforts should be made to achieve direct anastomosis of the bilateral cervical nerves during surgery. Univariate logistic analysis suggests that a shorter disease duration is associated with better prognosis, which aligns with the theory of the neural plasticity window period.^[40]^ Although multivariate analysis did not show significance, disease duration remains a key factor to be considered in clinical decision-making.

This study has several limitations. Its retrospective, single-center design introduces potential selection bias and precludes causal inference, necessitating future prospective, multicenter studies for validation. The sample size, while substantial for this specialized procedure, is modest for predictive modeling, and the model requires external validation in larger cohorts to confirm its generalizability. The absence of a control group limits our ability to isolate the effect of CC7 from natural recovery, and the limited 12-month follow-up data (n = 18) warrant cautious interpretation of long-term outcomes. Furthermore, the binary classification of rehabilitation adherence oversimplifies its role, and future studies should employ more granular metrics. The predictive power of our model is also limited by the absence of neuroimaging biomarkers (e.g., DTI), the integration of which is a critical future direction. Finally, the reliance on impairment-based scales over standardized assessments of activities of daily living or quality of life underscores the need for more patient-centered outcomes in future research. We did not adjust for multiple comparisons in the univariate analysis due to the exploratory nature of this study. While this increases the risk of false-positive findings, it was chosen to avoid excessive false negatives and generate hypotheses for future research. The results of the univariate analysis should therefore be interpreted with caution.

## 5. Conclusion

Contralateral C7 nerve transfer (CC7) effectively improves upper limb function in central spastic hemiplegia (60.3% efficacy rate; 45.2% FMA-UE improvement, *P* < .001) with a favorable safety profile (transient complications resolving within 6 months). Younger age (OR = 0.86) and higher baseline Fugl-Meyer score (OR = 1.45) are independent predictors of treatment success. The developed nomogram prediction model (AUC = 0.898) provides a practical tool for preoperative patient selection and personalized treatment planning.

## Author contributions

**Conceptualization:** Jiabo Xiao, Changzheng Dong.

**Data curation:** Jiabo Xiao.

**Formal analysis:** Jiabo Xiao, Shichang Guo.

**Investigation:** Jiabo Xiao, Shichang Guo, Binhong Li.

**Methodology:** Jiabo Xiao, Xiaohui Liu, Changzheng Dong.

**Project administration:** Changzheng Dong.

**Resources:** Bo Zhang, Changzheng Dong.

**Software:** Bo Zhang, Xiaohui Liu.

**Supervision:** Changzheng Dong.

**Writing – original draft:** Jiabo Xiao.

**Writing – review & editing:** Changzheng Dong.
